# Biocompatibility of HbV: Liposome-Encapsulated Hemoglobin Molecules-Liposome Effects on Immune Function

**DOI:** 10.3390/jfb8030024

**Published:** 2017-06-28

**Authors:** Hiroshi Azuma, Mitsuhiro Fujihara, Hiromi Sakai

**Affiliations:** 1 Department of Pediatrics, Asahikawa Medical University, Asahikawa 078-8510, Japan; 2 Japanese Red Cross, Hokkaido Block Blood Center, Sapporo 063-0802, Japan; fujihara@hokkaido.bc.jrc.or.jp; 3 Department of Chemistry, Nara Medical University, Kashihara 634-8521, Japan; hirosakai@naramed-u.ac.jp

**Keywords:** artificial red blood cell, hemoglobin, liposome, immune suppression, MDSC

## Abstract

Hemoglobin vesicles (HbVs) are oxygen carriers consisting of Hb molecules and liposome in which human hemoglobin (Hb) molecules are encapsulated. Investigations of HbV biocompatibility have shown that HbVs have no significant effect on either the quality or quantity of blood components such as RBC, WBC, platelets, complements, or coagulation factors, reflecting its excellent biocompatibility. However, their effects on the immune system remain to be evaluated. HbVs might affect the function of macrophages because they accumulate in the reticuloendothelial system. Results show that splenic T cell proliferation is suppressed after injection of not only HbV but also empty liposome into rat, and show that macrophages that internalized liposomal particles are responsible for the suppression. However, the effect is transient. Antibody production is entirely unaffected. Further investigation revealed that those macrophages were similar to myeloid-derived suppressor cells (MDSCs) in terms of morphology, cell surface markers, and the immune-suppression mechanism. Considering that MDSCs appear in various pathological conditions, the appearance of MDSC-like cells might reflect the physiological immune system response against the substantial burden of liposomal microparticles. Therefore, despite the possible induction of immunosuppressive cells, HbVs are an acceptable and promising candidate for use as a blood substitute in a clinical setting.

## 1. Introduction

Hemoglobin (Hb) based-oxygen carriers can be roughly categorized into two types: Acellular modified Hb molecules and cellular liposome-encapsulated Hb. The latter were developed by Waseda University, and were designated as Hb vesicles (HbVs), with phosphatidylcholine, cholesterol, PEG-conjugated lipid, a negative charged lipid, and concentrated Hb molecules. The mean particle size is 250 nm and the ζ potential is −18 mV [1,2]. They have sufficient oxygen transport capability equal to or greater than that of normal red blood cells. Moreover, HbVs are a promising blood substitute, suitable for clinical use [3].

The effect of HbV on human blood components such as polymorphonuclear cells, platelets, complement system, and coagulation system has been investigated since the prototype was developed. Results were summarized in an earlier report of the relevant literature [4]. No marked change in the number of PMN, platelets, RBC, human hematopoietic stem/progenitor cells, activity of CH50, or coagulation time was observed after administration of HbVs into a rat or ex vivo experimental setting. Furthermore, no marked change of the function of PMN and platelets was detected in an ex vivo experimental system [5,6]. These study results suggest that HbVs, including the most advanced type, have little, if any, interaction with these blood components, and therefore suggest that HbVs possess exclusively high biocompatibility.

The liposome used for current HbVs comprises 1,2-dipalmitoyl-*sn*-glycero-3-phosphatidylcholine(DPPC)/cholesterol (CHOL)/1,5-*O*-dihexadecyl-*N*-succinyl-l-glutamate(DHSG)/polyethylene glycol-conjugated1,2-distearoyl-sn-glycero-3-phosphatidylethanolamine (PEG5000-DSPE) at the molar ratio of 5:4:0.9:0.03 [6]. After administration, HbVs accumulate in the reticuloendothelial system (RES). It was demonstrated to be metabolized within a few days [7,8,9].

Generally speaking, little attention has been devoted to the effects of liposome on the immune system, possibly because the amount of liposome used as a drug delivery system is so small that it has no marked effect on the immune system in an experimental animal model. However, in a clinical setting, a considerable amount of HbVs must be transfused to substitute for red blood cells. For that reason, large numbers of liposomal microparticles are expected to be infused and be trapped by phagocytic cells in RES in the liver, spleen, and bone marrow. Consequently, it is possible that the function of phagocytic cells fluctuates, thereby changing the immune response. 

Our colleague has been examining this issue. The half-life of red blood cells is approximately 100 days in humans. In contrast, based on the half-life of HbV in cynomolgus monkeys (47–72 h) [8] that in humans is assumed to be shorter than 100 days. This means that more frequent transfusions of HbV are required to maintain blood Hb level high enough compared with red blood cell transfusion. The necessity of frequent injection itself is a problem, and it may accelerate the development of hemochromatosis. Therefore, HbV may not be a suitable blood substitute for the treatment of chronic anemia. However, in conditions where rapid blood transfusion is indispensable to save life, and there is no red blood cell product available to hand, injection of a large amount of HbV solution as a blood substitute must be the choice to save life. Therefore, in order to mimic this clinical situation, the injection of a large amount of liposome or (HbV) solution into rats was principally conducted only once to evaluate its effect on immune function.

This chapter presents a review of our data, and discusses possible effects of liposomal microparticles, including HbVs, on immune function.

## 2. Effects of Liposomal Microparticles on T Cell Proliferation

**Figure 1:**
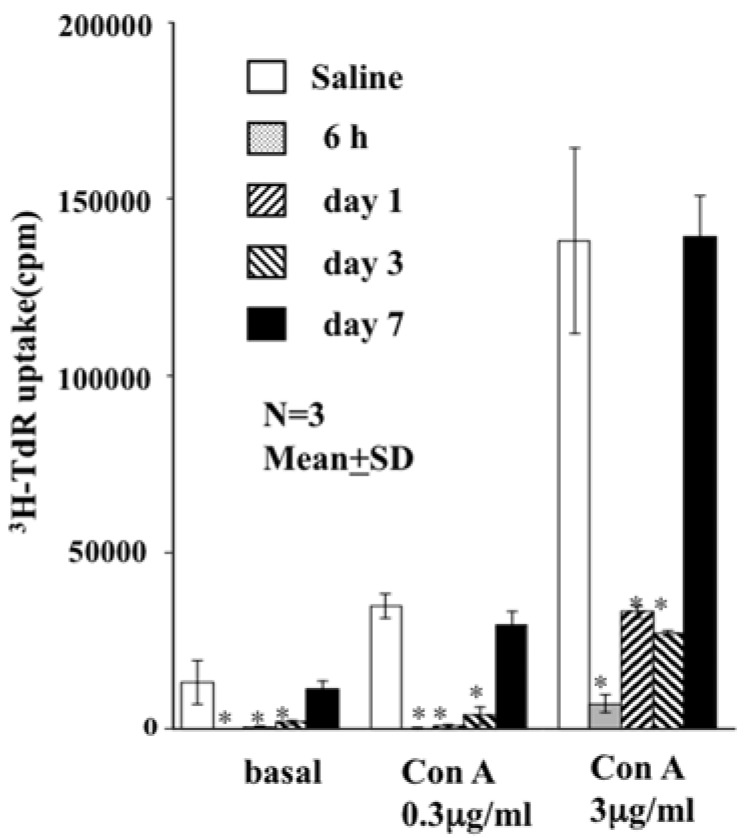
Effect of HbVs on proliferation of Con A-stimulated rat splenic T cells. 20% (*v*/*v*) of HbV solution was injected intravenously into rats. The spleen was excised 6 h to 3 days after injection. Then, the proliferative response of splenic T cells to Con A was evaluated. Compared to controls, T cell proliferation was inhibited from 6 h to 3 days after injection of HbVs (** *p* < 0.01). No suppression was observed after 7 days. (Figure modified from [10]).

The total blood volume of rats has been calculated as 56 mL/kg. Assuming a clinical setting, 20% (*v*/*v*) total blood volume of an HbV suspension (Hb concentration of approx. 10 g/dL) was injected into a W (kg) of the rat, which is equivalent to injection of 0.63 × W (g) of lipid. The spleen was excised later. Then, a single-cell suspension of splenocytes was cultured ex vivo in the presence of concanavalin A (Con A). Results show significant suppression of T cell proliferation compared to normal splenocytes [10]. The suppression was completely eliminated seven days after HbV injection (Figure 1).

In addition, results show that KLH-specific T cell proliferation (physiological T cell proliferation) was also suppressed. Evaluation of the expression of CD25 (a high-affinity IL-2 receptor) revealed that CD25 was expressed on Con A-stimulated T cells from both liposome-loaded and saline-loaded splenocytes (data not shown). Moreover, IL-2 was present in the culture supernatant of liposome-loaded splenocytes [10]. The amount of IL-2 produced was no less than that produced in control splenocytes. These results suggest that Con A stimulation can fully activate T cells in the liposome-loaded splenocyte culture, but that they are unable to proliferate. In other words, they enter a sort of anergic state. As described above, HbVs are particles composed of liposomal particles with Hb molecules in them. Therefore, the question of whether the liposome, Hb molecules, or both contribute this unexpected effect must be addressed.

**Figure 2:**
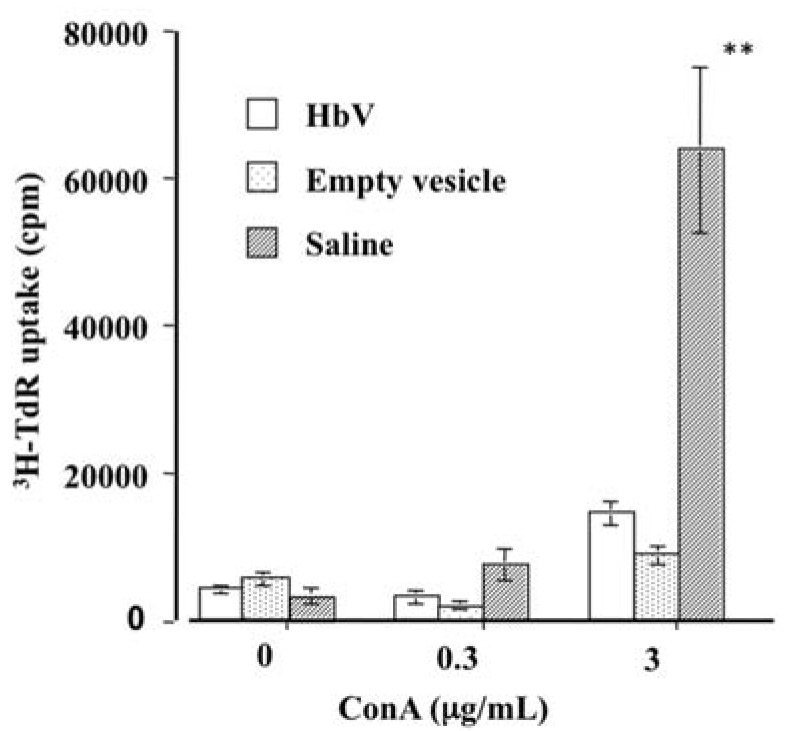
Effects of HbVs and Empty vesicles on proliferation of Con A-stimulated rat splenic T cells. Spleen was excised from rat 24 h after injection of HbVs, empty vesicle (liposome), or saline. Then splenocytes were stimulated with Con A. The proliferative response of splenic T cells derived from both HbV-loaded and empty vesicle-loaded rats were significantly lower than those of controls (** *p* < 0.01).

Results show that the suppression of T cell proliferation was observed even when empty vesicles were injected into rats (Figure 2).

Therefore, the liposome component, but not the Hb molecules within the liposome particles, caused the suppression of T cell proliferation.

**Figure 3:**
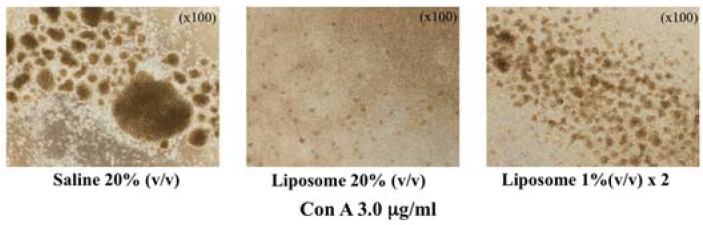
Inhibition of lymphocyte blastoid formation in liposome-loaded splenocytes. Even the lower dose of liposome (1% (*v*/*v*) × 2) can inhibit blastoid formation (cell proliferation).

In addition, suppression of T cell-blastoid formation by liposome injection was observed for a lower dose of liposome (Figure 3), indicating that this is not a toxic effect of large doses of liposome loading. It is actually a physiological response of the immune system against the liposomal microparticles.

## 3. Nitric Oxide Is the Effector Molecule for Suppression

**Figure 4:**
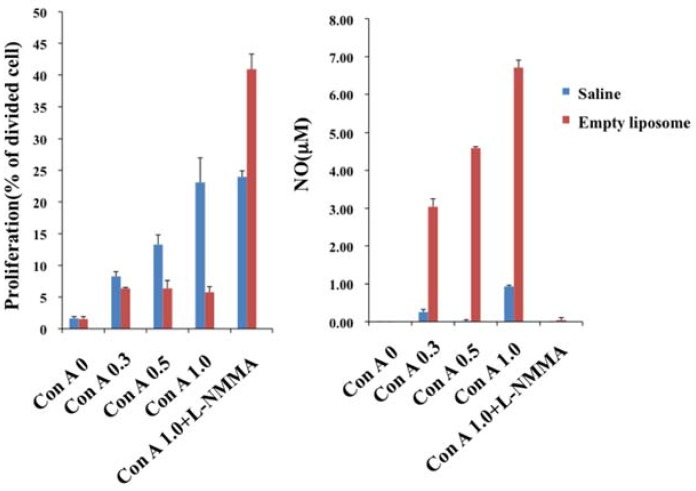
Correlation between inhibition of cell division and the production of NO. Splenocytes derived from liposome-loaded rat were cultured in the presence of Con A (mg/mL). Inhibition of T cell proliferation was visible for a wide range of Con A concentrations associated with enhanced production of NO. Inhibition of cell division was completely eliminated in the presence of iNOS inhibitor (L-NMMA, 2 mM).

Reportedly, nitric oxide (NO) production from macrophages is involved in the suppression of T cell proliferation [11,12,13]. We added inducible nitric oxide synthase (iNOS) inhibitor to the culture system, which revealed that the suppression of T cell proliferation was eliminated, and that production of NO in liposome-loaded splenocytes was suppressed completely compared with those in control splenocytes. This result demonstrated that NO is the main effector molecule for the suppression (Figure 4).

## 4. Cell-to-Cell Contact is Necessary

When normal splenocytes were cultured with liposome-loaded splenocytes in the presence of Con A, proliferation of whole T cells was suppressed. However, when they were cultured together but separated by a membrane, normal T cell proliferation was not suppressed [10]. This result suggests that close cell-to-cell contact is necessary for suppression.

## 5. Liposome-Internalized Macrophage is Responsible for T Cell Suppression

To identify the cells responsible for T cell suppression, liposome-internalized splenic macrophages were sorted using flow cytometry. Then, control splenocytes were stimulated by Con A in the presence of the sorted cells. Results show that liposome-internalized macrophages were responsible for T cell suppression [10]. At the same time, analysis of cell surface markers was conducted. Results showed that the liposome-internalized macrophages were positive for CD11b/c (OX42). Most of them were negative/weakly positive for class II and negative for CD80 and CD86.

## 6. Preferential Expression of iNOS Protein in Splenocytes Derived from Liposome-Loaded Rat

**Figure 5:**
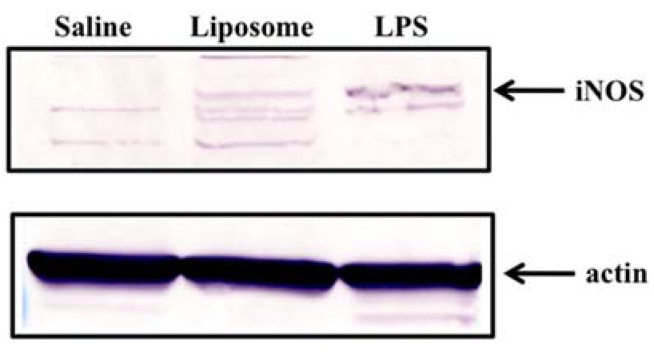
Induction of iNOS in the spleen after injection of liposome. iNOS became detectable in splenocyte lysate after liposome injection. As a positive control, splenocyte lysate after LPS injection was used.

To confirm the involvement of NO in the suppression, we addressed whether or not iNOS protein production is enhanced in the spleen after liposome injection. As expected, iNOS protein was detected only in liposome-loaded splenocytes (Figure 5).

In addition, iNOS protein was detected preferentially in the macrophage-rich fraction derived from liposome-loaded splenocytes [14]. Judging from these results, it is rational to speculate that proliferation of Con A-stimulated T cells was suppressed by NO produced by liposome-internalized macrophage and that the enhanced production of NO requires contact of liposome-internalized macrophage with activated T cells.

## 7. Dose-Dependent Suppressive Effects of Liposome-Internalized Cells

**Figure 6:**
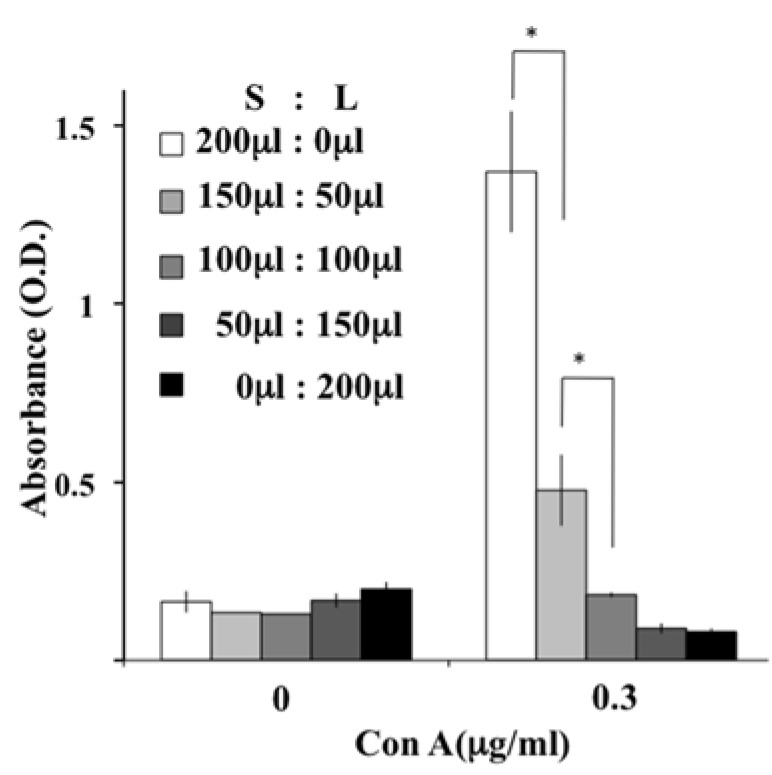
Dose-dependent inhibition effects of liposome-loaded splenocyte on T cell proliferation. Saline-loaded splenocyte suspension (S) was mixed with liposome-loaded splenocyte suspension (L) at the indicated volumes and was stimulated with Con A. The inhibition of T cell proliferation was enhanced as the volume of L increased (* *p* < 0.05).

When CD11b/c positive cells were removed from the liposome-loaded splenocytes, T cell proliferation was clearly restored [14], indicating that T cells in liposome-loaded splenocytes were intact, at least in terms of their proliferation ability. When saline-loaded splenocytes and liposome-loaded splenocytes were mixed at different ratios, T cell suppression was observed as the percentage of liposome-loaded splenocytes increased (Figure 6). Based on data indicating that T cells in liposome-loaded splenocytes are intact, liposome-internalized cells dose-dependently exert their suppressive effect on T cell proliferation.

## 8. Effects of HbVs on Antibody Production

**Figure 7:**
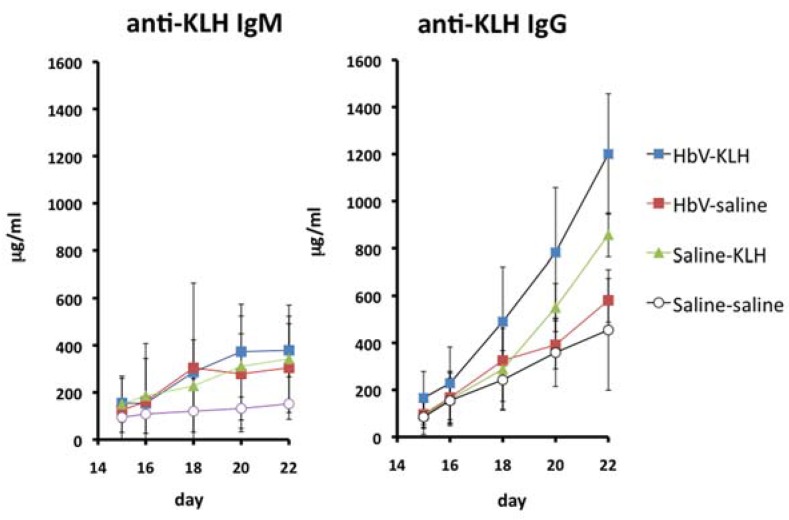
Antibody production against KLH was not influenced by HbV injection. Immunization with KLH was conducted under HbV injection into rat. Neither the primary response nor the secondary response was suppressed by the injection of HbVs into rats.

As described above, splenic T cell proliferation was transiently suppressed by liposome or HbVs. Therefore, the HbV effects on antibody production were addressed. Rats were immunized with keyhole limpet hemocyanin (KLH) with HbV injection. Then the anti-KLH antibody production was evaluated. Results showed that infusion of HbV interferes with neither primary nor secondary response (Figure 7) [15].

This result suggests that HbV immunotoxicity is negligible, at least in terms of antibody production.

## 9. Liposome Effects on Cytokine Production

As described above, IL-2 production from Con A-stimulated splenocytes was not suppressed by liposome-loading. We also addressed the production of IL-1β and IL-10, because macrophages are their main producer cells. Results showed that the production of IL-10 was enhanced. In contrast, the production of IL-1β was not influenced to any degree (Table 1).

Considering that IL-10 is an immunosuppressive cytokine, it might contribute to the suppression of T cell proliferation to a certain degree.

## 10. Similarity between Liposome-Internalized Cells and MDSC

Myeloid-derived suppressor cells (MDSC) are defined as immature cells derived from bone marrow myeloid precursors with the capability of suppressing T cell function. After they are induced in various pathological conditions, they suppress T cell immune response. They are classified into M-MDSC and PMN-MDSC [16,17]. The main features of the former are the following: They can suppress both antigen-specific and antigen-non-specific T cell proliferation; cell-to-cell contact is required for the suppression; the main effector molecule is NO; they express CD11b on their surface. It is noteworthy that the liposome-internalized cells meet these features. Enhanced production of IL-10 might not contradict this idea. Therefore, it can be speculated that transient conversion of macrophages into MDSC-like cells might occur when HbV or liposome is transfused intravenously into the body. However, it might not cause a marked adverse effect, because the effect is transient. Moreover, antibody production is unaffected.

The possible induction of MDSC-like cells by liposome or HbVs is an unexpected pharmacological effect. As mentioned above, HbVs (or liposomes) are negatively charged microparticles. Recently, negatively charged microparticles (500 nm in size) have been reported to induce T cell tolerance or suppress inflammatory response by phagocytic cells that take up the microparticles [18,19]. Some macrophages take up negatively charged microparticles through a scavenger receptor; the macrophage receptor with a collagenous structure (MARCO). This leads to the induction of T cell tolerance, sequestration of microparticle-internalized macrophages in the spleen, etc. Considering that the liposomes used in our experiments are 250 nm in diameter with negative ζ potential, it can be speculated that macrophages expressing scavenger receptors such as MARCO uptake intravenously-administered liposomes. As a result, they accumulate in the spleen and turn into MDSC-like cells capable of T cell suppression. Therefore, investigation how liposomes are taken up by macrophages may be warranted. Furthermore, considering that MDSCs are expected to work as a negative regulator for immune reactions, exploring the underlying molecular mechanism of macrophage transition to MDSC-like cells by liposome might contribute to the development of more ideal HbV or new drugs having immunosuppressive effects.

## 11. Conclusions

HbV might induce immunosuppressive cells in vivo. Nevertheless, results have shown that the effects are transient and that the production of specific antibody in vivo was not influenced at all. These results, coupled with our earlier data suggesting HbVs do not interact with the blood components, lead us to infer that HbVs are acceptable as a blood substitute for clinical use. Furthermore, our data indicating that immunosuppressive cells transiently induced by HbV or liposome particles closely resemble MDSCs challenge us to find new immunomodulators.

## Figures and Tables

**Table 1:** Fold increase of cytokine production.

	IL-10		IL-1β	
	Saline	Liposome	Saline	Liposome
Exp. 1	3	6.3	1.1	1.2
Exp. 2	2.7	5	0.7	0.8
Exp. 3	2.4	4.5	0.8	1.2

Fold increase of IL-10 was significantly higher in liposome-loaded splenocytes than in saline-loaded splenocytes. In contrast, no difference of fold increase was found for IL- β production.
